# The Association Between Reactive Oxygen Metabolites and Metabolic Syndrome in Asymptomatic Japanese Men

**DOI:** 10.4021/jocmr668w

**Published:** 2011-09-26

**Authors:** Kazuhiko Kotani, Nobuyuki Taniguchi

**Affiliations:** a Department of Clinical Laboratory Medicine, Jichi Medical University, Tochigi, Japan

## Abstract

**Background:**

The association between the oxidative status and metabolic syndrome (MetS) should be studied in various populations with various oxidative stress-related markers. The aim of this cross-sectional study was to investigate the association between oxidative status, as assessed by the reactive oxygen metabolites (d-ROMs) test, and MetS in asymptomatic Japanese men, in relation to age.

**Methods:**

The serum d-ROMs levels were measured in cardiovascular disease-free, non-smoking, non-medicated males (n = 140), who were divided into groups as follows: Group 1, < 60 years (n = 75, mean age 46 ± 9 [SD] years), and Group 2, ≥ 60 years (n = 65, mean 68 ± 6 years). The MetS was determined by the NCEP-ATP recommendations with minor modifications for a Japanese population.

**Results:**

There was no significant difference in the d-ROMs levels between the subjects with and without MetS in Group 2 (≥ 60 years), but the subjects with MetS (n = 38, 324 ± 59 U. Curr.) exhibited significantly higher d-ROMs levels than those without MetS (n = 37, 290 ± 49 U. Curr., P < 0.01) in Group 1 (< 60 years). These differences did not change even after adjustments for basic confounders.

**Conclusions:**

These results suggest that oxidative status, as assessed by the d-ROMs, can be enhanced among asymptomatic younger, but not older, Japanese males with MetS. Further studies are required to establish the observed associations.

**Keywords:**

Oxidative stress; Reactive oxygen species; D-ROMs; Obesity; Metabolic syndrome

## Introduction

When cardiometabolic risk factors such as obesity, hyperglycemia, dyslipidemia and hypertension are clustered, cardiovascular disease (CVD) risks are remarkably increased [1]. While metabolic syndrome (MetS) represents a clustering disorder of such risk factors, a deeper understanding of the underlying mechanisms of the contribution of MetS to CVD is needed to control CVD events [2]. One of the major pathophysiologies of MetS is an oxidative condition caused by the overproduction and inactivation of reactive oxygen species (ROS) [3]. Although numerous studies have examined oxidative status in MetS subjects using oxidative stress-related markers, these results are not always consistent [4-12]. This can partly be due to differences in not only the studied populations but also in the markers used; namely, we note the weaker associations in healthier relative to diseased populations [4,7,11] and a different significance of markers used on MetS traits is reported even in the same population [10]. Recently, the reactive oxygen metabolites (d-ROMs, Diacron, Italy) test was developed to quantify oxidative status by measuring the hydroperoxidation of organic compounds (occurring in lipids, proteins, nucleic acids, etc.) [13,14], and is also used in medical settings [15-17]. However, the literature on determining oxidative status using this test in MetS subjects is rare.

Another debatable point is a difference in the clinical relevance of MetS on CVD between genders and age; namely, there may be a higher CVD risk in men with MetS than women [18] and minimal MetS-CVD linkage in relatively older populations [12,19,20]. Therefore, the aim of this study was to investigate the association between the levels of d-ROMs and MetS properties in an asymptomatic Japanese male population, in considering different age groups.

## Methods

A total of 140 non-smoking, non-medicated male participants were recruited during routine check-ups in health education classes and outpatient clinics. This population was divided into two groups by age: Group 1, < 60 years of age and Group 2, ≥ 60 years of age (Table 1). Eligible subjects had no histories of cardiovascular, thyroid, hematological, renal, hepatic, or collagenous diseases. The presence of MetS was diagnosed with at least 3 out of 5 of the following criteria according to the National Cholesterol Education Program Adult Treatment Panel (NCEP-ATP) III recommendations [2]: 1) obesity identified by body mass index (BMI) ≥ 25.0 kg/m2 as a surrogate for a Japanese population [21]; 2) increased blood pressure (BP) identified by systolic BP (SBP) ≥ 130 mmHg and/or diastolic BP (DBP) ≥ 85 mmHg; 3) hypertriglyceridemia identified by serum triglyceride (TG) ≥ 1.69 mmol/L; 4) low high-density lipoprotein cholesterol (HDL-C) identified by serum HDL-C < 1.04 mmol/L, and 5) hyperglycemic status identified by a fasting plasma glucose (FPG) ≥ 6.1 mmol/L. The study was approved by the institutional ethics committee, and all subjects gave their informed consent.

**Table 1:** Clinical Characteristics Between Groups 1 (< 60 Years) and 2 (≥ 60 Years)

**Variable**	**Group 1 (n = 75)**	**Group 2 (n = 65)**	**P-value**
Age (years)	46 ± 9	68 ± 6	< 0.01**
Body mass index (kg/m^2^)	25.6 ± 3.6	24.0 ± 2.9	< 0.01**
Systolic BP (mmHg)	135 ± 19	147 ± 23	< 0.01**
Diastolic BP (mmHg)	83 ± 12	82 ± 11	0.84
Glucose (mmol/L)	6.71 ± 2.54	7.25 ± 2.48	0.21
LDL-cholesterol (mmol/L)	3.85 ± 1.40	3.75 ± 0.79	0.58
Triglycerides (mmol/L)	1.84 (1.40 - 3.62)	1.99 (1.32 - 2.64)	0.14
HDL-cholesterol (mmol/L)	1.42 ± 0.42	1.44 ± 0.37	0.80
D-ROMs (U. Carr.)	307 ± 57	327 ± 65	0.06

BP, blood pressure; LDL, low-density lipoprotein; HDL, high-density lipoprotein; d-ROMs, reactive oxygen metabolites. Statistical significance (by unpaired t-test between Groups 1 and 2): ** P < 0.01.

In addition to BMI, the SBP and DBP levels were determined in the seated subject’s right-arm with a mercury sphygmomanometer. Blood was sampled after an overnight fast with no alcohol consumption or exercise during the preceding day. The serum TG and FPG levels were measured using enzymatic methods, and the serum low-density lipoprotein (LDL-C) and HDL-C levels were measured using homogeneous methods (Sekisui Co. Ltd., Tokyo, Japan). The d-ROMs values were obtained using a kinetic spectrophotometric assay (F.R.E.E system; Diacron, Italy) with the intra- and inter-assay coefficients of variation of 2.1% and 3.1%, respectively [13,14]. Briefly, serum samples were mixed with a buffered solution, and a chromogenic substrate was added into the mixture. The mixture was centrifuged and then incubated in the thermostatic block of the system. The absorbance was then recorded at 505 nm. Measurements are expressed as U. Carr., and 1 U. Carr. corresponds to 0.08 mg/dL H_2_O_2_ (the reference value is suggested to be approximately 250 - 300 U. Carr.).

Data are expressed as the means ± SD or medians plus interquartile range. Differences between two groups were analyzed using the unpaired *t*-test. The influence of MetS (as a fixed variable) on d-ROMs (as a dependent variable) was examined using a general linear model analysis with adjustments for basic confounders (i.e., age or LDL-C that is not included in the diagnostic criteria of MetS but can contribute to the development of CVD and oxidative status). The values of TG were log-transformed for the analyses because of the skewed distribution. A P-value of < 0.05 was considered to be statistically significant.

## Results

The clinical characteristics between age-stratified groups are shown in Table 1. Group 2 (≥ 60 years) showed significantly higher age and SBP levels and a lower BMI level than Group 1 (< 60 years). Although Group 2 had high FPG and d-ROMs levels relative to Group 1, these differences did not reach statistically significant levels.

The prevalence of MetS was 51% and 57% in Groups 1 and 2, respectively, as shown in Table 2. There were no significant differences in the d-ROMs levels between each criterion of MetS in either group (Table 2). However, while there was no significant difference in the d-ROMs levels between the subjects with and without MetS in Group 2 (P = 0.26), the subjects with MetS exhibited a significantly higher d-ROMs level than those without MetS in Group 1 (P < 0.01). The difference in the d-ROMs levels by MetS remained statistically significant, even after adjusting for age (F = 7.46, P < 0.01) and for age and LDL-C (F = 11.11, P < 0.01).

**Table 2:** Comparative Data on the d-ROMs Levels Between the Absence and Presence of Metabolic Syndrome and its Criteria in Groups 1 (< 60 Years) and 2 (≥ 60 Years)

**Criteria**	**Group 1 (n = 75)**		****	**Group 2 (n = 65)**	
	**absence (n)**	**presence (n)**	****	**absence (n)**	**presence (n)**
Obesity	298 ± 59 (36)	316 ± 53 (39)		330 ± 72 (40)	323 ± 52 (25)
Increased BP	304 ± 56 (26)	309 ± 57 (49)		302 ± 58 (13)	333 ± 65 (52)
Hyperglycemia	306 ± 63 (47)	308 ± 44 (28)		317 ± 56 (26)	334 ± 70 (39)
Hypertriglyceridemia	297 ± 47 (25)	312 ± 60 (50)		322 ± 68 (26)	331 ± 63 (39)
Hypo-HDL-cholesterolemia	304 ± 54 (67)	336 ± 74 (8)		329 ± 65 (60)	301 ± 56 (5)
Metabolic syndrome	290 ± 49 (37)	324 ± 59 (38)**		317 ± 69 (28)	335 ± 60 (37)

Comparative Data on the d-ROMs Levels Between the Absence and Presence of Metabolic Syndrome and its Criteria in Groups 1 (< 60 Years) and 2 (≥ 60 Years) d-ROMs, reactive oxygen metabolites; BP, blood pressure; HDL, high-density lipoprotein. Statistical significance (by unpaired t-test between the absence and presence of metabolic syndrome and its criteria in Groups 1 and 2): ** P < 0.01.

## Discussion

The present study revealed a significantly higher d-ROMs level in Japanese male subjects with MetS than those without MetS in Group 1 (< 60 years), but not Group 2 (≥ 60 years). It would therefore be valuable to add new findings to our current knowledge regarding the association between MetS and oxidative status, by using the d-ROMs test that differed from the test described in previous studies [4-12].

The first notable finding of this study is confirmation that, although no significant differences in the d-ROMs levels were observed between each criterion of MetS, MetS clearly expressed the hyperoxidative status of the d-ROMs. This is in agreement with the clustering concept of MetS (a disorder clustering of each criterion) to remarkably increase the CVD risks [1,2]. Fat and systemic ROS, which stem from obese adipocytes and unfavorable lifestyles, are associated with the augmentation of NADPH-oxidase and suppression of antioxidative enzymes, and these pathways can directly lead to the initiation/development of MetS via cell damage and indirectly via dysregulated adipocytokines [3,22]. The progression/exacerbation of the MetS critera further promotes a vicious circle with the addition of oxidative stress induced at atherosclerotic sites [3]. The biological mechanisms can account for the present finding of a significant increase of the d-ROMs levels in MetS subjects. In addition, although the previous studies suggested a significant increase of the d-ROMs in hypertensive subjects [15] and obese subjects [17], weak differences in the d-ROMs between solo criterion groups observed in the present study might partially be explained by the differences in studied populations (the present study population was of a relatively good state and/or mild state, even though the subjects showed the diseased levels, as seen in Table 1).

The second notable finding of this study is confirmation that a significant increase of the d-ROMs levels in MetS was clearly observed in the younger Group 1, rather than the older Group 2. Previous studies indicated weak magnitudes of MetS on CVD in elderly populations [19,20], while another study reported a significant positive relationship between MetS and oxidative status in an elderly population [12]. Although the precise reasons for these findings remain to be unclear, our present study seems to support the former studies [19,20]. Because aging *per se* is an oxidative condition (in fact, Group 2 tended to show somewhat high d-ROMs levels relative to Group 1, as seen in Table 1) [23], it may be difficult or confusing to distinguish clearly the oxidative status between pathologies in older subjects. This seems to be an important view to control MetS-related CVD events, if the potential magnitudes of MetS on CVD can differ by age. The possibility that the influence of MetS on CVD can be modulated by age merits further investigations.

The present study had a few limitations. The cross-sectional design did not allow for determining the cause-and-effect relationship. The study was conducted in male participants only and in relatively small sample sizes. Future studies with larger populations including females and prospective/interventional designs are needed.

In summary, the present study showed significantly higher d-ROMs levels in asymptomatic Japanese male subjects with MetS than those without MetS in Group 1 (< 60 years), but not Group 2 (≥ 60 years). The oxidative status can be enhanced under the clustering concept of MetS in younger men in particular, which may imply an age-modulated effect of MetS on CVD. Further studies are necessary to establish the observed associations.
